# Is obesity associated with emotional and behavioural problems in children? Findings from the Millennium Cohort Study

**DOI:** 10.3109/17477166.2010.526221

**Published:** 2011-06

**Authors:** Lucy J Griffiths, Carol Dezateux, Andrew Hill

**Affiliations:** 1 MRC Centre of Epidemiology for Child Health, UCL Institute of Child HealthLondon, UK; 2 Academic Unit of Psychiatry & Behavioural Sciences, Institute of Health Sciences, Leeds University School of MedicineLeeds, UK

**Keywords:** Children, emotional and behavioural problems, mental health, obesity, prospective relationships, Strengths and Difficulties, Questionnaire

## Abstract

*Objectives*. We examined cross-sectional and longitudinal associations between obesity and emotional and behavioural problems in a nationally representative sample of young children. *Methods*. Data were available from 11 202 children (50% boys) participating in the UK's Millennium Cohort Study. Height and weight were measured at 3 and 5 years and children defined as obese using IOTF cut-offs for body mass index (BMI). Emotional and behavioural problems were parentally assessed using the Strengths and Difficulties Questionnaire. Adjusted linear and multinomial regression analyses were conducted separately for boys and girls. *Results*. At age 3, obese boys had more conduct problems, and obese girls had more prosocial behaviours, than their normal weight counterparts. At age 5, obese boys had more conduct problems, hyperactivity and inattention problems, peer relationship problems and total difficulties. Obese girls only had more peer relationship problems. Obese 3-year-olds were not at increased risk of abnormal scores; in contrast, obese 5-year-old boys were significantly more likely to have abnormal scores for conduct problems, hyperactivity and inattention problems, peer relationship problems, prosocial behaviours and total difficulties. Obesity, at age 3, was also predictive of peer relationship problems at age 5 in boys (95% CI: 0.26 [0.01, 0.52]). *Conclusions*. Childhood obesity is associated with emotional and behavioural problems from a very young age. Obese boys are at particular risk. Further research is required to examine effect modifiers and mediating factors in these associations. Recognition and response to these mental health problems should be a goal of pediatric obesity interventions and policies.

## Introduction

Childhood obesity continues to be a worldwide public health problem, and one that is occurring at ever younger ages (1, 2). Data from the National Health and Nutrition Examination Survey (NHANES) (2003–2006) indicates the prevalence of obesity is 12.4% in children aged 2–5 years (3). Within the European Union, the reported prevalence of obesity at 4 years ranges from 2.8% in the Netherlands (2002–2006) to 15.5% in Spain (2006) (4). As action to address obesity increases, so does interest in the psychological consequences of this disease.

Several studies have been published examining associations between children's overweight and a wide range of mental health problems, including self-esteem (5–7), depression (8), quality of life (7, 9), body dissatisfaction (10) and other emotional and behavioural problems (11–17). Findings from these studies are inconsistent in the reported associations of psychological impairment with obesity. We have previously suggested that focusing on obesity, rather than combining overweight and obese children in these analyses, is one way of improving clarity in this relationship (7).

Younger age groups have received far less research attention in this area, despite preschool years being identified as a crucial time for obesity prevention programmes (18) and psychosocial problems included as prime targets of these interventions (19). Further evidence is therefore needed to inform understanding of relationships between obesity and mental health problems, the age of occurrence and whether early obesity signals the likelihood of problems at an older age. Large longitudinal cohort studies are well-placed to provide this.

Accordingly, this study examined associations between obesity and emotional and behavioural problems in preschool aged children. Specifically, we used prospectively collected data on obesity and emotional and behavioural problems, collected at ages 3 and 5, in a nationally representative cohort study to explore cross-sectional and longitudinal associations. In addition to exploring obese-normal differences, this study examined risk of abnormal symptom scores for obese children. Given evidence of gender differences in other areas of obesity-related psychological impairment at older ages (e.g., self-esteem and depression) with females at greater risk (20), gender-stratified analyses were conducted. It was hypothesized that obese children would have more problems than normal weight children, obese children would be more likely to have abnormal symptom scores, and that there would be greater psychological impairment in obese girls than boys.

## Methods

### Subjects and design

The Millennium Cohort Study (MCS) is a prospective study of the social, economic and health-related circumstances of children born in the United Kingdom (UK) at the start of the new century. The sample was drawn from births between September 2000 and January 2002 who were resident in the UK and eligible for Child Benefit (a universal benefit for families with children). Children living in disadvantaged areas, from ethnic minority groups and from Wales, Scotland and Northern Ireland were over-represented by using a stratified clustered sampling design to ensure the cohort was nationally representative.

The first contact with the cohort was at 9 months, when information was collected on 18 818 infants (18 296 singletons); 72% of those approached (21). The second and third surveys took place when the cohort were aged 3 and 5 years (22). Each contact involved home visits where interviews were conducted with main (usually the child's mother) and partner respondents. The MCS received ethical approval from the South West and London Multi-Centre Research Ethics Committees, UK.

In these analyses we examined data from the first, second and third contacts, obtained from the UK Data Archive, University of Essex, UK. Seventy-one percent (12 989/18 296) of the singleton infants from the original cohort participated at all three contacts. From these 12 989 singletons, families were excluded if there were two cohort children from the same family [7], the main survey respondent was not the child's biological mother [216], the mother's partner was not male [198], if the child had a missing or implausible body mass index (BMI) at age 3 or 5 [1 083 and 190, respectively], or if they had missing information for any of the emotional or behavioural problems at age 3 [908] or age 5 [467]. Some participants had more than one exclusion criterion, resulting in a final sample size of 10 837 children for the age 3 cross-sectional analyses, and 11 202 children for the age 5 cross-sectional and the longitudinal analyses.

### Predictor variables (weight status at age 3 and 5 years)

At age 3 and 5, trained interviewers weighed the children, without shoes or outdoor clothing, using Tanita HD-305 scales (Tanita UK Ltd., Middlesex, UK), and weights were recorded in kilograms to one decimal place. Heights were measured with the Leicester Height Measure Stadiometer (Seca Ltd., Birmingham, UK) and recorded to the nearest millimetre. Childhood overweight and obesity, at both ages, was defined using the International Obesity Task Force (IOTF) cut-offs for BMI (23). Those below the overweight cut-off were classified as ‘normal weight’.

### Outcome measures (emotional and behavioural problems)

At age 3 and 5 years, parents were asked to complete the Strengths and Difficulties Questionnaire (SDQ) (24). The SDQ is a 25-item emotional and behavioural screening questionnaire that examines: emotional, conduct, hyperactivity and inattention, and peer relationship problems, plus pro-social behaviour. Scale scores range from 0 to 10. Higher scores on the first four of these subscales and lower scores on the pro-social subscale indicate more problems. A ‘total difficulties’ score is generated by summing the scores from all scales except the prosocial scale. The resultant score ranges from 0 to 40 and higher scores indicate more difficulties. In addition, the SDQ instructions (24) provide bandings for categorization as ‘normal’, ‘borderline’ or ‘abnormal’ in order to identify children who are likely ‘cases’ with mental health disorders. This study adopted both approaches.

The SDQ is reported to have high test-retest reliability and good validity (25), and has been used in other large epidemiological studies, such as the British Child Mental Health Survey and the Avon Longitudinal Study of Parents and Children (26, 27).

### Confounding factors

Social, demographic and health information was collected on the cohort child and their families at the first, second and third contact. Confounding factors considered in this study included: the child's ethnicity (categorised according to guidelines from the Office for National Statistics (28)), the mother's socio-economic circumstances (categorised according to the National Statistics Socio-economic Classification (29)) and highest academic qualification attained, maternal age at the MCS birth, whether the mother was a lone parent, the number of children in the household, the household income, and whether the mother was receiving any treatment for doctor-diagnosed depression or serious anxiety. Finally, mothers reported their height and weight, and were defined as overweight or obese if their BMI was ≥25 kg/m^2^, or ≥30 kg/m^2^, respectively. These factors were adjusted for in the analyses as they were either found to be significantly associated with both the predictor (weight status) and outcome (SDQ subscales) variables within the models, or because previous literature has identified them as being potential confounding factors.

For the purpose of the longitudinal analyses (described below), two additional factors were controlled for: emotional/behavioural problems at age 3, and weight status at age 5.

### Statistics

Analyses were conducted in STATA/SE 10.0 (Stata Corporation, TX), using survey commands to allow for the cluster sampling design and to obtain robust standard errors. Weighted percentages, univariate and adjusted analyses were calculated using survey and non-response weights to account for the sampling design and attrition between contacts. P values were calculated by adjusted Wald tests.

Cross-sectional associations between weight status and emotional and behavioural problems at ages 3 and 5 were investigated using adjusted linear regression analyses. Analyses were completed for all children (data not shown) and as obesity-gender interactions were found for some of the emotional and problem behaviours (e.g., peer relationships and total difficulties), the analyses were stratified by gender and repeated. Using the categorised SDQ variables at both ages, adjusted multinomial regression analyses were also conducted to explore if obese children were more likely to have abnormal scores.

Longitudinal associations between weight status at age 3 and the emotional and behavioural problems at age 5 were also investigated using linear regression analyses. Problems at age 3 were associated with problems at age 5; similarly, weight status at age 3 was associated with weight status at age 5 (data not shown); therefore, to isolate the longitudinal effect of weight status at age 3 on the likelihood of having emotional and behavioural problems at age 5, we controlled for the confounding factors listed above (level A of adjustment), the child's prior problems at age 3 (level B of adjustment) and current weight status at age 5 (level C of adjustment).

While regression coefficients are provided for the overweight groups, these results are not discussed as this study focused, *a priori*, on obese-normal weight differences.

## Results

### Sample descriptives

At age 3, 77% of the children were normal weight (boys: 78%; girls: 77%) and 4.9% were obese (boys: 4.4%; girls: 5.5%). At age 5, 79% were normal weight (boys: 81%; girls: 77%) and 5.2% were obese (boys: 4.7%; girls: 5.6%).

Table I displays the characteristics of the 5-year-olds included in this study and, separately, of the normal weight and obese 5-year-olds.

**Table I:** Sample characteristics at age 5

	Weighted % (n)		
	
	NW (8797)	Obese (601)	All (11202)
**Socio-demographic characteristics**			
Child's sex*			
Boys	40.7 (4 574)	2.4 (277)	50.2 (5 653)
Girls	38.3 (4 223)	2.8 (324)	49.9 (5 549)
Child's ethnicity*			
White	70.9 (7 657)	4.3 (499)	89.5 (9 767)
Mixed	2.2 (224)	0.2 (21)	2.9 (290)
Asian	3.8 (618)	0.3 (45)	4.7 (746)
Black	1.2 (166)	0.3 (30)	2.0 (246)
Other ethnic group	0.9 (114)	0.1 (4)	1.0 (130)
Maternal socio-economic circumstances*			
Managerial and professional occupations	26.0 (2 749)	1.3 (147)	32.5 (3 441)
Small employers and own account workers	3.3 (331)	0.2 (20)	4.2 (416)
Intermediate occupations	15.3 (1 628)	0.9 (91)	18.8 (2 018)
Lower supervisory and technical occupations	4.3 (493)	0.3 (36)	5.4 (626)
Semi-routine and routine occupations	26.0 (2 913)	2.0 (239)	33.7 (3 827)
Never worked and long-term unemployed	4.2 (595)	0.4 (59)	5.3 (754)
Maternal highest academic qualification*			
Degree	15.8 (1 729)	0.6 (68)	19.1 (2 093)
Diploma in higher education	7.8 (858)	0.5 (54)	9.9 (1 088)
A/AS/S levels	8.1 (914)	0.3 (43)	10.0 (1 139)
O levels/GCSE grades A—C	28.3 (3 017)	2.0 (225)	36.0 (3 902)
GCSE grades D—G	7.9 (861)	0.7 (76)	10.5 (1 136)
Other academic qualifications	1.5 (189)	0.2 (17)	1.9 (235)
None of these qualifications	9.7 (1 216)	0.9 (116)	12.7 (1 590)
Age at MCS birth (years)*			
Mean (SD)	28.9 (5.8)	29.2 (5.8)	28.9 (5.8)
Lone motherhood status**			
Non-lone mother	65.9 (7 314)	4.1 (479)	82.8 (9 253)
Lone mother	13.2 (1 483)	1.0 (122)	17.2 (1 949)
Number of children in the household**			
1	12.2 (1 348)	1.0 (120)	16.0 (1 805)
2 or 3	39.6 (4 281)	2.5 (280)	49.9 (5 427)
4 or more	27.3 (3 168)	1.7 (201)	34.1 (3 970)
Household income**			
£13 000 per annum	15.4 (1 844)	1.2 (148)	19.7 (2 381)
£13 000–26 000 per annum	25.8 (2 972)	1.8 (215)	32.9 (3 806)
£26 000–36 400 per annum	15.3 (1 641)	1.0 (121)	19.7 (2 146)
£36 400+ per annum	22.7 (2 277)	1.1 (114)	27.7 (2 792)
**Maternal heath characteristics**			
Maternal treatment for depression or anxiety**			
No	73.3 (8 125)	4.7 (543)	92.5 (10 311)
Yes	5.7 (672)	0.5 (58)	7.5 (890)
Maternal weight status**			
Normal weight	50.4 (4 630)	1.8 (177)	59.3 (5 497)
Overweight	19.0 (1 853)	1.5 (152)	25.2 (2 476)
Obese	10.4 (1 042)	1.7 (164)	15.4 (1 530)

NW: Normal weight.

* Information collected at first MCS contact (9 months).

** Information collected at third MCS contact (5 years).

Missing (all children): sex 0; ethnicity 23; maternal socio-economic status 120; maternal highest qualification 19; age at MCS birth 2; lone motherhood status 0; no. siblings in household 0; household Income 77; Maternal treatment for depression or anxiety 1; Maternal weight status 1 699.

### Cross-sectional associations between obesity and emotional and behavioural problems at age 3 and 5

At age 3, obese boys had significantly higher mean scores for conduct problems, hyperactivity and inattention problems, peer relationship problems and total difficulties, than normal weight boys (Table II). Compared with normal weight girls, obese girls had higher mean scores for prosocial behaviour, indicating that they had *more* prosocial behaviours. At age 5, obese boys had significantly higher mean scores for emotional problems, conduct problems, hyperactivity and inattention problems, peer relationship problems and total difficulties, than normal weight boys. At the same age and compared with normal weight girls, obese girls had higher mean scores for peer relationship problems and total difficulties.

**Table II:** Mean (standard deviation) Strengths and Difficulties Questionnaire scores at age 3 and 5

	Boys			Girls		
		
SDQ scores	NW	Obese	All	NW	Obese	All
**Age 3** (n = 10 837)						
Emotional symptoms	1.3 (1.4)	1.3 (1.6)	1.3 (1.4)	1.3 (1.5)	1.2 (1.3)	1.3 (1.4)
Conduct problems	2.8 (2.0)	3.2 (2.2)*	2.8 (2.1)	2.6 (2.0)	2.8 (1.9)	2.6 (2.0)
Hyperactivity/Inattention problems	4.0 (2.4)	4.4 (2.5)*	4.1 (2.4)	3.5 (2.2)	3.8 (2.4)	3.5 (2.3)
Peer problems	1.5 (1.6)	1.8 (1.7)*	1.5 (1.6)	1.3 (1.4)	1.5 (1.4)	1.3 (1.4)
Prosocial behaviour	7.2 (1.9)	7.1 (1.9)	7.1 (1.9)	7.5 (1.8)	7.8 (1.6)*	7.6 (1.8)
Total difficulties	9.6 (5.2)	10.8 (5.9)*	9.8 (5.2)	8.8 (4.9)	9.3 (4.9)	8.8 (4.9)
**Age 5** (n = 11 202)						
Emotional symptoms	1.2 (1.5)	1.5 (1.6)*	1.2 (1.5)	1.4 (1.5)	1.4 (1.5)	1.4 (1.5)
Conduct problems	1.5 (1.5)	2.0 (1.7)*	1.6 (1.5)	1.3 (1.4)	1.4 (1.4)	1.3 (1.4)
Hyperactivity/Inattention problems	3.5 (2.4)	4.3 (2.8)*	3.5 (2.4)	2.9 (2.2)	3.0 (2.4)	2.9 (2.2)
Peer problems	1.1 (1.4)	1.6 (1.8)*	1.1 (1.4)	1.0 (1.2)	1.4 (1.5)*	1.0 (1.3)
Prosocial behaviour	8.2 (1.7)	8.0 (1.9)	8.2 (1.7)	8.7 (1.5)	8.7 (1.5)	8.7 (1.5)
Total difficulties	7.4 (4.9)	9.4 (5.9)*	7.5 (5.0)	6.5 (4.4)	7.3 (4.8)*	6.5 (4.5)

* Significant difference (p≤0.05) between normal weight (NW) and obese groups.

[Lower scores on the pro-social subscale indicate more problems].

Linear regression, controlling for potential confounders, confirmed that, at age 3, obese boys had more conduct problems, and obese girls had more prosocial behaviours than their normal weight counterparts (Table III). At age 5, obese boys had more conduct problems, hyperactivity and inattention problems, peer relationship problems and total difficulties. Obese girls only had more peer relationship problems.

**Table III:** Cross-sectional associations between weight status and Strengths and Difficulties Questionnaire scores at age 3 and 5. Adjusted regression coefficients and 95% confidence intervals

	N	Emotional	Conduct	Hyperactivity	Peer	Prosocial	Total
**Age 3**							
*Boys*							
NW	4 229	0	0	0	0	0	0
Overweight	978	−0.23 (−0.34, −0.12)*	0.24 (0.06, 0.42)*	0.22 (−0.02, 0.43)*	0.01 (−0.13, 0.14)	−0.17 (−0.33, −0.01)*	0.24 (−0.19, 0.67)
Obese	254	−0.14 (−0.39, 0.11)	0.35 (0.01, 0.69)*	0.10 (−0.34, 0.54)	0.17 (−0.11, 0.45)	0.11 (−0.25, 0.47)	0.48 (−0.46, 1.42)
*Girls*							
NW	4 058	0	0	0	0	0	0
Overweight	1 025	−0.05 (−0.16, 0.06)	−0.10 (−0.26, 0.07)	−0.03 (−0.23, 0.16)	−0.05 (−0.16, 0.07)	0.14 (−0.03, 0.31)	−0.23 (−0.64, 0.18)
Obese	293	−0.09 (−0.29, 0.12)	−0.03 (−0.31, 0.24)	−0.01 (−0.31, 0.28)	−0.01 (−0.22, 0.20)	0.37 (0.10, 0.64)*	−0.14 (−0.81, 0.53)
**Age 5**							
*Boys*							
NW	4 574	0	0	0	0	0	0
Overweight	802	−0.10 (−0.23, 0.03)	0.06 (−0.09, 0.21)	0.21 (−0.03, 0.45)	−0.03 (−0.16, 0.11)	0.08 (−0.08, 0.24)	0.15 (−0.33, 0.62)
Obese	277	0.15 (−0.09, 0.38)	0.38 (0.14, 0.61)*	0.68 (0.27, 1.08)*	0.38 (0.08, 0.68)*	−0.15 (−0.45, 0.15)	1.58 (0.72, 2.44)*
*Girls*							
NW	4 223	0	0	0	0	0	0
Overweight	1 002	−0.16 (−0.30, −0.02)*	−0.06 (−0.17, 0.06)	−0.00 (−0.18, 0.18)	0.00 (−0.12, 0.12)	0.08 (−0.05, 0.22)	−0.22 (−0.61, 0.16)
Obese	324	−0.12 (−0.32, 0.07)	−0.02 (−0.23, 0.19)	−0.16 (−0.51, 0.19)	0.21 (0.02, 0.41)*	0.16 (−0.04, 0.36)	−0.08 (−0.72. 0.56)

* P ≤ 0.05 compared with referent (normal weight [NW]).

Adjusted for child's ethnicity, maternal socio-economic status, mother's highest academic qualification, maternal age at MCS birth, household income, lone-motherhood status, no. of children in the household, maternal treatment for depression or anxiety, maternal weight status.

The adjusted multinomial regression analyses indicated that, at age 3, there were no significant differences in obese boys’ or girls’ likelihood of having abnormal scores on any of the SDQ scales. In contrast, at age 5, significant differences were found. Obese boys were more likely to have abnormal scores for conduct problems (relative risk ratio [95% CI]: 1.7 [1.1, 2.7], p = 0.03), hyperactivity and inattention problems (1.9 [1.3, 2.9], p = 0.001), peer relationship problems (2.3 [1.4, 3.9], p = 0.002), prosocial behaviours (2.5 [1.2, 5.3], p = 0.01) and total difficulties (2.3 [1.3, 4.1], p = 0.01). In this age group there were not enough obese girls with abnormal scores to calculate risk.

### Longitudinal associations between obesity at age 3 and problems at age 5

In adjusted longitudinal analyses (Table IV), obese 3-year-old boys had more conduct problems and total difficulties at age 5 than normal weight boys, even after controlling for socio-demographic and maternal characteristics (level A of adjustment). However, once adjustment was made for prior problems at age 3 (level B of adjustment), and then also weight status at age 5 (level C of adjustment), these associations attenuated and became non-significant. In contrast, obese 3-year-old boys had more peer relationship problems at age 5 across all levels of adjustment. For girls, obesity at age 3 was not significantly associated with any emotional or behavioural problems at age 5.

**Table IV:** Longitudinal associations between weight status at age 3 and Strengths and Difficulties Questionnaire scores at age 5. Adjusted regression coefficients and 95% confidence intervals

	Emotional	Conduct	Hyperactivity	Peer	Prosocial	Total
**Level A of adjustment**						
*Boys*						
NW	0	0	0	0	0	0
Overweight	−0.07 (−0.02, 0.06)	0.11 (−0.03, 0.25)	0.13 (−0.10, 0.35)	0.03 (−0.09, 0.15)	0.03 (−0.12, 0.17)	0.20 (−0.25, 0.66)
Obese	−0.04 (−0.30, 0.22)	0.31 (0.05, 0.56)*	0.40 (−0.03, 0.84)	0.44 (0.17, 0.71)*	−0.01 (−0.32, 0.30)	1.11 (0.20, 2.01)*
*Girls*						
NW	0	0	0	0	0	0
Overweight	−0.18 (−0.29, −0.07)*	−0.08 (−0.18, 0.03)	−0.16 (−0.34, 0.01)	−0.01 (−0.12, 0.09)	0.09 (−0.04, 0.21)	−0.44 (−0.78, −0.09)*
Obese	0.02 (−0.22, 0.26)	0.02 (−0.21, 0.24)	−0.03 (−0.41, 0.35)	0.11 (−0.10, 0.31)	0.14 (−0.05, 0.33)	0.12 (−0.60, 0.83)
**Level B of adjustment**						
*Boys*						
NW	0	0	0	0	0	0
Overweight	0.02 (−0.10, 0.13)	0.03 (−0.09, 0.16)	0.03 (−0.16, 0.21)	0.04 (−0.08, 0.16)	0.11 (−0.03, 0.24)	0.09 (−0.29, 0.47)
Obese	−0.00 (−0.24, 0.23)	0.19 (−0.03, 0.41)	0.13 (−0.20, 0.46)	0.33 (0.08, 0.57)*	−0.02 (−0.30, 0.26)	0.64 (−0.06, 1.34)
*Girls*						
NW	0	0	0	0	0	0
Overweight	−0.14 (−0.24, −0.03)*	−0.03 (−0.13, 0.06)	−0.09 (−0.25, 0.07)	−0.00 (−0.10, 0.10)	0.04 (−0.08, 0.16)	−0.22 (−0.52, 0.07)
Obese	0.04 (−0.19, 0.26)	−0.03 (−0.23, 0.17)	−0.07 (−0.42, 0.28)	0.08 (−0.11, 0.27)	0.04 (−0.14, 0.22)	−0.01 (−0.62, 0.60)
**Level C of adjustment**						
*Boys*						
NW	0	0	0	0	0	0
Overweight	0.02 (−0.10, 0.15)	0.02 (−0.11, 0.15)	−0.10 (−0.29, 0.09)	0.06 (−0.07, 0.18)	0.09 (−0.05, 0.24)	−0.02 (−0.40, 0.36)
Obese	−0.09 (−0.34, 0.16)	0.10 (−0.15, 0.35)	0.03 (−0.36, 0.42)	0.26 (0.01, 0.52)*	0.03 (−0.25, 0.31)	0.16 (−0.58, 0.91)
*Girls*						
NW	0	0	0	0	0	0
Overweight	−0.09 (−0.21, 0.03)	−0.03 (−0.13, 0.07)	−0.09 (−0.30, 0.09)	−0.04 (−0.15, 0.07)	0.03 (−0.10, 0.17)	−0.24 (−0.57, 0.09)
Obese	0.13 (−0.15, 0.41)	−0.03 (−0.26, 0.21)	−0.00 (−0.40, 0.39)	−0.04 (−0.25, 0.18)	−0.00 (−0.25, 0.25)	0.01 (−0.70, 0.71)

* P ≤ 0.05 compared with referent (normal weight [NW]).

Adjustments: Level A: child's ethnicity, maternal socio-economic status, mother's highest academic qualification, maternal age at birth, household income, lone-motherhood status, no. of children in the household, maternal treatment for depression or anxiety, maternal weight status; Level B: level ‘A’ plus prior problems at age 3; Level C: level ‘A’ plus prior problems at age 3 and weight status at age 5.

## Discussion

This study has shown the emergence of an association between obesity, and emotional and behavioural problems in very young children. At age 3, obesity was associated with conduct problems in boys, while obese girls displayed more prosocial behaviours. However, obesity was associated with a greater range of problems at age 5, suggesting a strengthening of the relationship. At this age, obese boys had more conduct problems, hyperactivity and inattention problems, peer relationship problems, and total difficulties. Increased risk of abnormal scores at this age also captured the clinical, not simply statistical, meaningfulness of these associations. Contrary to our hypothesis, obese girls displayed less psychological impairment as this weight status was only associated with peer relationship problems. Finally, by using the longitudinal nature of the data, we have shown that obesity at age 3 was associated with risk of peer relationship problems in boys at age 5, even after controlling for their early emergence and current weight.

Most previous research in this area has used cross-sectional study designs. In general, while older obese children (from approximately 8 years of age) and adolescents display more impaired well-being compared with normal weight counterparts (11–15), the results of studies investigating younger children are more varied (11, 12, 16, 17). For example, Lawlor et al. (11) found that overweight was not associated with behavioural problems at age 5, in boys or girls. However, their study combined overweight and obese groups and so did not focus on obese children. While Drukker et al. (12) also failed to report significant associations between obesity and SDQ scores, analyses were carried out on a combined sample of both boys and girls. Given that we found very few problems in obese girls, aggregating data may have led to reductions in effect sizes. In contrast, other cross-sectional studies have reported evidence of psychological problems (16, 17). Indeed, our findings most closely resemble those of Sawyer et al. (17) who used the SDQ in a nationally representative sample of Australian children. Obese 4 to 5-year-old boys had more conduct problems and total difficulties (teacher-reported, but not parent-reported) than normal weight boys. Obesity was also only associated with peer relationship problems in girls, similar to our cross-sectional findings at age 5.

Our observation of a relationship between early obesity and later peer relationship problems in boys contrasts with the null findings of two other longitudinal datasets (11, 16). These differences in outcome may be related to the instruments used to measure total behavioural problems. By using the SDQ and examining behavioural problems independently (i.e., the subscales of total difficulties) we have explored which of these were (peer problems) and were not (e.g., conduct problems) longitudinally associated with obesity. Additionally, the 9-year gap in one of the studies (11) may have been too long to establish a prospective association.

Our study has a number of strengths, in addition to reporting on cross-sectional and longitudinal associations. The sample was large and drawn from a contemporary, nationally representative, cohort of young children. Weight and height was measured using trained interviewers and standardised protocols. The analysis adjusted for a wide range of factors, including an indicator of the mental and physical health status of the cohort child's mother. Furthermore, the parent-proxy report version of the SDQ is a valid and reliable instrument for assessing a range of mental health problems in children (30), and the clinical relevance of our findings is shown through the ability of the SDQ to assess abnormal scores. Nevertheless, it is important to acknowledge that parents completed the SDQ, and have been observed to overestimate other psychological problems in obese children compared with children's self-assessments (7). Parents of obese children are also more likely to be overweight or obese themselves than parents of normal weight children (31). Consequently, their own weight history and recollection of childhood may impact on how they parent their own children and how sensitive they are to their children's experiences. As a consequence, triangulation of views from parents, teachers and, at an older age, from children may provide a more complete picture of the psychological morbidities associated with child obesity.

The scale items themselves give clues about the behaviours that mothers are reporting on behalf of their obese sons. Hyperactivity scale items ask about restlessness and distractibility. Conduct scale items include temper tantrums, dishonesty, and bullying other children. The peer problems scale asks about solitary play and being bullied. The overall description is of boys more likely to be seen as unsettled, erratic, physical in some peer relationships, and excluded from others. Some of these qualities may be exaggerations of gendered stereotypes of boy's behaviour and reflect different parenting styles used by their mothers. Others may be behavioural consequences following stigmatisation by peers, parents or even educators. Aggressive coping strategies have been observed in slightly older obese children (33). Impaired peer relationships in both boys and girls are consistent with observations on victimisation. For example, we have previously shown that obesity is predictive of victimisation in preadolescent children, using data from a different UK longitudinal cohort (32). These findings therefore emphasize the necessity for carers of young obese children, particularly boys, who are experiencing emotional or behavioural problems, to be extra vigilant of difficult social experiences that may be contributing to these problems.

Further research is needed to examine effect modifiers of the associations found in our study. For example, initiatives within schools to reduce stigmatisation towards obese youth (34) may help to protect obese boys from friendship problems. Additional attention should be paid to potential mediators, such as low self-confidence, which can result from obesity and can also be a barrier to developing new peer relationships (33). Relationships in the opposite direction also need examination, to evaluate whether emotional and behavioural problems are risk factors for obesity. Such associations have been reported in adolescents and the idea of a bi-directional relationship between obesity and affective disorder is gaining momentum (35). Similar complex conceptualisations should not be restricted to older age groups.

Overall, this research is a valuable reminder of how early relationships between obesity and well-being can emerge. If psychological problems are a barrier to treatment uptake and adherence (36), then action should follow. Accepting this justifies strategies that recognise and reduce psychological distress in obese youth, within both community and clinical settings.

## Funding

Lucy Griffiths was initially supported by a MRC Special Training Fellowship in Health Services and Health of the Public Research (grant G1061221) and then by the Wellcome Trust (grant 084686/Z/08/A). The Centre for Paediatric Epidemiology and Biostatistics is supported in part by the Medical Research Council in its capacity as the MRC Centre of Epidemiology for Child Health. Research at the UCL Institute of Child Health and Great Ormond Street Hospital for Children receives a proportion of the funding from the Department of Health's National Institute for Health Research Biomedical Research Centres funding scheme. The Millennium Cohort Study is funded by grants to Professor Health Joshi, Director of the study, from the Economic and Social Research Council and a consortium of government funders. The study sponsors played no part in the design, data analysis and interpretation of this study, the writing of the manuscript, or the decision to submit the paper for publication and the authors’ work was independent of their funders.

## References

[1] Jotangia D, Moody A, Stamatakis EWH (2007). Obesity among children aged under 11.

[2] Kim J, Peterson KE, Scanlon KS (2006). Trends in overweight from 1980 through 2001 among preschool-aged children enrolled in a health maintenance organization. Obesity.

[3] Centers for Disease Control and Prevention http://www.cdc.govobesitychildhoodprevalence.html.

[4] Cattaneo A, Monasta L, Stamatakis E (2010). Overweight and obesity in infants and pre-school children in the European Union: a review of existing data. Obes Rev.

[5] Miller CT, Downey KT (1999). A meta-analysis of heavyweight and self-esteem. Pers Soc Psychol Rev.

[6] French SA, Story M, Perry CL (1995). Self-esteem and obesity in children and adolescents: a literature review. Obes Res.

[7] Griffiths LJ, Parsons TJ, Hill AJ (2010). Self-esteem and quality of life in obese children and adolescents: a systematic review. Int J Pediatr Obes.

[8] Britz B, Siegfried W, Ziegler A (2000). Rates of psychiatric disorders in a clinical study group of adolescents with extreme obesity and in obese adolescents ascertained via a population based study. Int J Obes Relat Metab Disord.

[9] Tsiros MD, Olds T, Buckley JD (2009). Health-related quality of life in obese children and adolescents. Int J Obes (Lond).

[10] Wadden TA, Foster GD, Stunkard AJ (1989). Dissatisfaction with weight and figure in obese girls: Discontent but not depression. Int J Obes (Lond).

[11] Lawlor DA, Mamun AA, O'Callaghan MJ (2005). Is being overweight associated with behavioural problems in childhood and adolescence? Findings from the Mater-University study of pregnancy and its outcomes. Arch Dis Child.

[12] Drukker M, Wojciechowski F, Feron FJ (2009). A community study of psychosocial functioning and weight in young children and adolescents. Int J Pediatr Obes.

[13] Gibson LY (2008). Clustering of psychosocial symptoms in overweight children. Aust N Z L Psychiatry.

[14] BeLue R, Francis LA, Colaco B (2009). Mental health problems and overweight in a nationally representative sample of adolescents: effects of race and ethnicity. Pediatrics.

[15] Lumeng JC, Gannon K, Cabral HJ (2003). Association between clinically meaningful behavior problems and overweight in children. Pediatrics.

[16] Datar A, Sturm R (2004). Childhood overweight and parent- and teacher-reported behavior problems: evidence from a prospective study of kindergartners. Arch Pediatr Adolesc Med.

[17] Sawyer MG, Miller-Lewis L, Guy S (2006). Is there a relationship between overweight and obesity and mental health problems in 4- to 5-year-old Australian children?. Ambul Pediatr.

[18] Hawkins SS, Law C (2006). A review of risk factors for overweight in preschool children: a policy perspective. Int J Pediatr Obes.

[19] van Wijnen LG, Wendel-Vos GC, Wammes BM (2009). The impact of school-based prevention of overweight on psychosocial well-being of children. Obes Rev.

[20] Wardle J, Cooke L (2005). The impact of obesity on psychological well-being. Best Pract Res Clin Endocrinol Metab.

[21] Plewis I (2007). Millennium Cohort Study: Technical Report on Sampling.

[22] Hansen K (2008). Millennium Cohort Study First, Second, and Third Surveys: A Guide to the Datasets.

[23] Cole TJ, Bellizzi MC, Flegal KM (2000). Establishing a standard definition for child overweight and obesity worldwide: International survey. BMJ.

[24] Youthinmind http://www.sdqinfo.com.

[25] Goodman R (1997). The Strengths and Difficulties Questionnaire: a research note. J Child Psychol Psychiatry.

[26] Goodman R, Ford T, Simmons H (2000). Using the Strengths and Difficulties Questionnaire (SDQ) to screen for child psychiatric disorders in a community sample. Br J Psychiatry.

[27] Wiles NJ, Peters TJ, Heron J (2006). Fetal growth and childhood behavioral problems: results from the ALSPAC cohort. Am J Epidemiol.

[28] Office for National Statistics (2003). Ethnic Group Statistics: A Guide for the Collection and Classification of Ethnicity Data.

[29] Rose D, Pevalin D (2003). A Researcher's Guide to the National Statistics Socio-economic Classification.

[30] Goodman A, Goodman R (2009). Strengths and difficulties questionnaire as a dimensional measure of child mental health. J Am Acad Child Adolesc Psychiatry.

[31] Hawkins SS, Cole TJ, Law C (2009). An ecological systems approach to examining risk factors for early childhood overweight: findings from the UK Millennium Cohort Study. J Epidemiol Community Health.

[32] Griffiths LJ, Wolke D, Page AS (2006). Obesity and bullying: different effects for boys and girls. Arch Dis Child.

[33] Griffiths LJ, Page AS (2008). The impact of weight-related victimization on peer relationships: the female adolescent perspective. Obesity (Silver Spring).

[34] Puhl RM, Latner JD (2007). Stigma, obesity, and the health of the nation's children. Psychol Bull.

[35] Goodman E, Whitaker RC (2002). A prospective study of the role of depression in the development and persistence of adolescent obesity. Pediatrics.

[36] Zeller M, Kirk S, Claytor R (2004). Predictors of attrition from a pediatric weight management program. J Pediatr.

